# GRNbenchmark - a web server for benchmarking directed gene regulatory network inference methods

**DOI:** 10.1093/nar/gkac377

**Published:** 2022-05-24

**Authors:** Deniz Seçilmiş, Thomas Hillerton, Erik L L Sonnhammer

**Affiliations:** Department of Biochemistry and Biophysics, Stockholm University, Science for Life Laboratory, Box 1031, 17121 Solna, Sweden; Department of Biochemistry and Biophysics, Stockholm University, Science for Life Laboratory, Box 1031, 17121 Solna, Sweden; Department of Biochemistry and Biophysics, Stockholm University, Science for Life Laboratory, Box 1031, 17121 Solna, Sweden

## Abstract

Accurate inference of gene regulatory networks (GRN) is an essential component of systems biology, and there is a constant development of new inference methods. The most common approach to assess accuracy for publications is to benchmark the new method against a selection of existing algorithms. This often leads to a very limited comparison, potentially biasing the results, which may stem from tuning the benchmark's properties or incorrect application of other methods. These issues can be avoided by a web server with a broad range of data properties and inference algorithms, that makes it easy to perform comprehensive benchmarking of new methods, and provides a more objective assessment. Here we present https://GRNbenchmark.org/ - a new web server for benchmarking GRN inference methods, which provides the user with a set of benchmarks with several datasets, each spanning a range of properties including multiple noise levels. As soon as the web server has performed the benchmarking, the accuracy results are made privately available to the user via interactive summary plots and underlying curves. The user can then download these results for any purpose, and decide whether or not to make them public to share with the community.

## Graphical Abstract

**Graphical Abstract:**
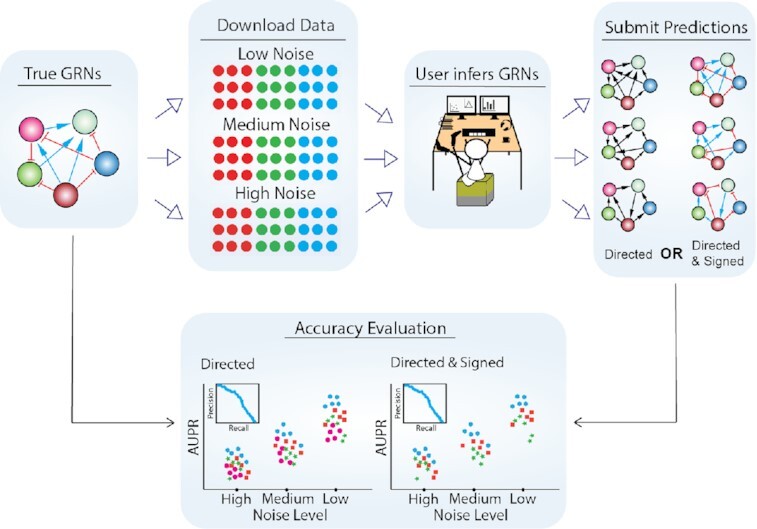
GRNbenchmark is a novel web server for online benchmarking of gene regulatory network inference methods. It offers easy and comprehensive benchmarking of new methods as well as comparison to a range of public methods. Gene expression datasets generated from "true" GRNs are downloaded by the user who infers GRNs and submits these back to the web server which calculates prediction accuracies and visualizes the results.

## INTRODUCTION

Gene regulatory networks (GRNs) are key to understanding physiological and pathological mechanisms in organisms, and therefore their accurate inference can identify novel mechanisms and treatment targets for genetic diseases (1–3). For these reasons, a large number of methods have been developed to infer GRNs from measurements of gene expression (4–7).

A common approach when proposing a new inference method is to benchmark it against a number of previously published methods to be able to assess its accuracy in comparison to others for a broader view of the method's contribution to the field (6,7). However, this comes with a risk that the selection of benchmarking data may introduce biases that could favor the new method, for instance by representing properties that are biologically unrealistic. Furthermore, if the authors of a new method devise a new benchmark, they need to rerun previous methods to compare to, which is often challenging and may lead to erroneous results if they are not run correctly.

The DREAM challenges (8–10) have attempted to solve this problem to a degree by providing standardized benchmarks based on a large amount of benchmarking data, which has become the most preferred approach in the field to evaluate a method's accuracy in comparison to other methods. Even though this was a great advance in the field, it is limited in terms of evaluating accuracy as a function of a range of data properties, such as noise levels, which has previously been shown to strongly affect inference accuracy (11–16).

In addition to these limitations of previous benchmarks, it can also be difficult and arduous to ensure that they are used properly. For instance, very few benchmarks present the ROC or PR curves that underlie the area under receiver-operating-characteristic and precision-recall curves (AUROC and AUPR values, respectively), and if the curves are not inspected, errors due to *e.g*. undetected truncation or mislabeling may occur. It is also valuable to inspect the underlying curves in order to understand the data produced by the method, to identify which regions it is most accurate in.

A solution to all these issues could be achieved with a standardized online framework where the benchmarking procedure is automated, eliminating biases and other problems. An example from another field is the online framework for orthology benchmarking (17), yet no such online service exists in the GRN inference field. The online orthology benchmark is widely used and has stimulated the development of many new orthology detection algorithms (18), and serves many purposes in that community (19,20).

Here we present GRNbenchmark, an online benchmarking framework inspired by the aforementioned orthology benchmark service but adapted to the needs of the GRN inference community. It provides developers with a large collection of datasets with varied properties for inferring GRNs with their own method, and performs automated benchmarking, visualization, and reporting of the accuracy results together with the publicly available ones in a publication-appropriate format. This way, GRNbenchmark solves several big challenges in the field. It also serves as a guide for users of published GRN inference methods, to help them select the most suitable method.

## MATERIALS AND METHODS

**Figure 1.**
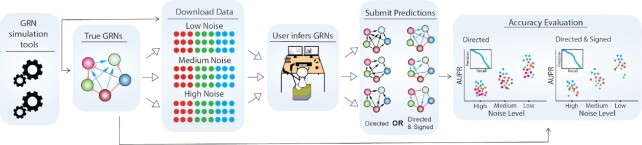
The workflow of GRNbenchmark. The diagram shows the entire process, starting from generation of benchmark data to GRN inference by a user to benchmarking and visualization. The rightmost panel represents the web server which accepts submissions and calculates prediction accuracies and visualizes them.

The workflow of GRNbenchmark is illustrated in Figure 1. Given a benchmark consisting of true GRNs, gene expression data generated from the true GRNs, and a set of GRNs inferred by a user, GRNbenchmark will apply the benchmarking procedure described below and visualize the results in both overview plots and in great detail. The web server was written in the R programming language 4.2.1 (https://www.R-project.org/) and is based on Dash 0.9.3 (https://dash.plotly.com/r) and a GraphQL engine (hasura/graphql-engine:v2.1.1) on top of postgres:14 as database backend. The entire web server is deployed in a Docker 20.10.12 container.

### Data generation, true GRNs and data

The current version of GRNbenchmark provides benchmarking datasets generated using two different simulation tools: GeneNetWeaver (21) and GeneSPIDER (11). Each tool was used to generate 5 true GRNs, from which gene expression datasets of three noise levels were generated.

#### Generation via GeneNetWeaver

Five subnetworks of 100 genes with directed and signed edges were extracted from the *E. coli* network available at GeneNetWeaver. The number of regulators in these networks varies between 85–86. The vertices were drawn by the option ‘random’, and the edges were assigned by the ‘greedy’ option. The sparsity of the five GRNs is 1.93 interactions per gene on average. Autoregulation is permitted in the true GRNs since they are important for the system's stability but such links are not considered in calculating the sparsity and accuracy because the main purpose of GRN inference is to identify regulatory interactions between genes and the inference accuracy should represent this. For each of the five GRNs, a noise-free gene expression dataset was generated from steady-state knockdown perturbations from ordinary differential equations (ODEs). No normalization was performed, and noise-free fold changes were calculated by the base 2 logarithm ratio between the gene expression and wild type. The fold change matrix was transposed to have genes on rows and experiments on columns, and replicated three times, representing replicated perturbation experiments in size 100 × 300 (genes x experiments).

#### Generation via GeneSPIDER

Five scale-free networks with directed and signed (activation or inhibition) edges were generated, allowing autoregulation (for the same reasons as for GeneNetWeaver GRNs), each being a network of only regulator genes. Three interactions per gene on average were assigned to each GRN, and the sparsity of these five GRNs, without selfloops, range between 2.22 and 2.39 interactions per gene on average. For each true GRN, a noise-free fold change gene expression dataset with three replicates of steady-state knockdown experiments was generated. GeneSPIDER also applies an ODE model for gene expression data generation. The resulting noise-free fold change gene expression matrix is in size 100 × 300 (genes x experiments).

#### Noise generation

For datasets from both GeneNetWeaver and GeneSPIDER, Gaussian noise matrices from three different levels, high, medium, and low, were generated from the required standard deviation estimated following Eq. 1 for signal-to-noise ratios (SNRs) 0.01, 0.1, and 1, respectively.

(1) $$\begin{equation*}\sigma {\rm{\ }} = \frac{{min\left( {svd\left( X \right)} \right)}}{{SNR\sqrt {{\chi ^{ - 2}}\left( {\alpha ,{\rm{\ }}NM} \right)} }}\ \end{equation*}$$

In Eq. 1, $svd( X )$ represents the vector of singular values from the singular value decomposition of the noise-free fold change gene expression matrix *X*; *α* is the type-I error, 0.05; *N* and *M* are the number of genes and experiments, respectively. $\sigma$ refers to the standard deviation.

### Publicly available methods

Eight well-known GRN inference methods: Least squares, LASSO (5), Ridge regression, ElasticNet (22), Z-score (23), GENIE3 (4), PLSNET (7), and TIGRESS (6), were run and made public on GRNbenchmark. The algorithm parameters used to infer GRNs using these inference methods are provided in the ‘Public Methods’ page on GRNbenchmark.

### Benchmarking

The benchmarking is performed by comparing the users’ inferred GRNs to the true GRNs that were used to generate the corresponding datasets that users inferred GRNs from. True positives are defined as predicted links connecting genes in the same direction as in the true GRN for the unsigned benchmark, and for the signed benchmark they also have to have the same sign. Selfloops are ignored. All other predicted links are defined as false positives. True negatives are links missing in both the predicted and true GRN. The accuracy of the inferred GRNs in reconstructing the underlying true GRN is measured in terms of three metrics: area under precision recall (AUPR), area under receiver-operating characteristic (AUROC), and the maximum F1-score. The first two measures, AUPR and AUROC, span the entire range of sparsities, *i.e*. the number of interactions per gene on average. Some inference methods such as least squares and Z-score infer fully connected GRNs, where the interactions can be sorted from the strongest to weakest. Some methods like Genie3 may infer fully connected or sparser GRNs depending on parameter selection. Penalty-based methods such as LASSO and Ridge regression infer GRNs for given regularization parameters, resulting in a GRN with a certain sparsity. By taking subsets of top ranked interactions from fully connected GRNs, complete precision-recall and ROC curves can be obtained, leading to AUPR and AUROC values that are comparable within the range of [0,1]. However, to be able to obtain and compare AUPR and AUROC values in the [0,1] range for GRNs that are not fully connected, we applied an extrapolation strategy, where the incomplete curves are made complete based on the probabilities of inferring a true interaction for the remaining part of the curve, the same way as was done in DREAM5 (8).

### Plotting methods

All plots are generated in the R programming language by the *ggplot2* package v3.3.5 (24) and made interactive by the *plotly* package v4.10.0 (25) only for the overview scatter. For other functionalities of the plots and guidelines to better understand the results, we refer the reader to the help page provided on the web server.

## RESULTS

GRNbenchmark is an online benchmarking tool for GRN inference methods. It currently provides two different benchmarks from two tools, GeneNetWeaver and GeneSPIDER, each containing fifteen 100-gene datasets for five networks and three noise levels. The framework supports addition of more benchmarks from other tools, network sizes, and data properties such as noise levels, meaning the benchmarks can be continuously improved by the authors based on advances in science and user feedback.

GRNbenchmark has four main functionalities: downloading data, submitting predictions, performing benchmarking, and viewing the benchmark results.

### Downloading data

Clicking on the ‘Download Data’ button downloads the benchmark data in a compressed file. Each dataset has a gene expression data and a perturbation design matrix, which are provided in separate files, in total 60 files. File names are intended to be self-explanatory to trace which tool (GeneNetWeaver or GeneSPIDER), network, data property (noise level), and data type (gene expression or perturbation) they correspond to.

#### The user infers GRNs

Once the datasets are downloaded, the user infers GRNs with their own method. For each benchmark, GRNs for all datasets must be inferred. The user can submit predictions for one or multiple benchmarks, but benchmarking will only be performed for complete sets.

### Submitting predictions

All files together – the inferred GRNs for all datasets (currently 15 per data generation tool) and tools (GeneNetWeaver and/or GeneSPIDER), should be bundled in a single compressed file for submission to GRNbenchmark. Each inferred GRN is stored as a separate CSV file for which detailed format instructions are provided on the submission page, as well as an example file. The web server then performs a quality check for the submitted files such as whether the files were named as requested, if the GRNs follow the correct format, and if only correct gene names are included. GRNbenchmark will then proceed with complete network sets only. For example if all files for the GeneSPIDER benchmark were available but missing files for the GeneNetWeaver benchmark were detected, GRNbenchmark will perform the benchmarking only for the GeneSPIDER benchmark.

### Benchmarking

**Figure 2.**
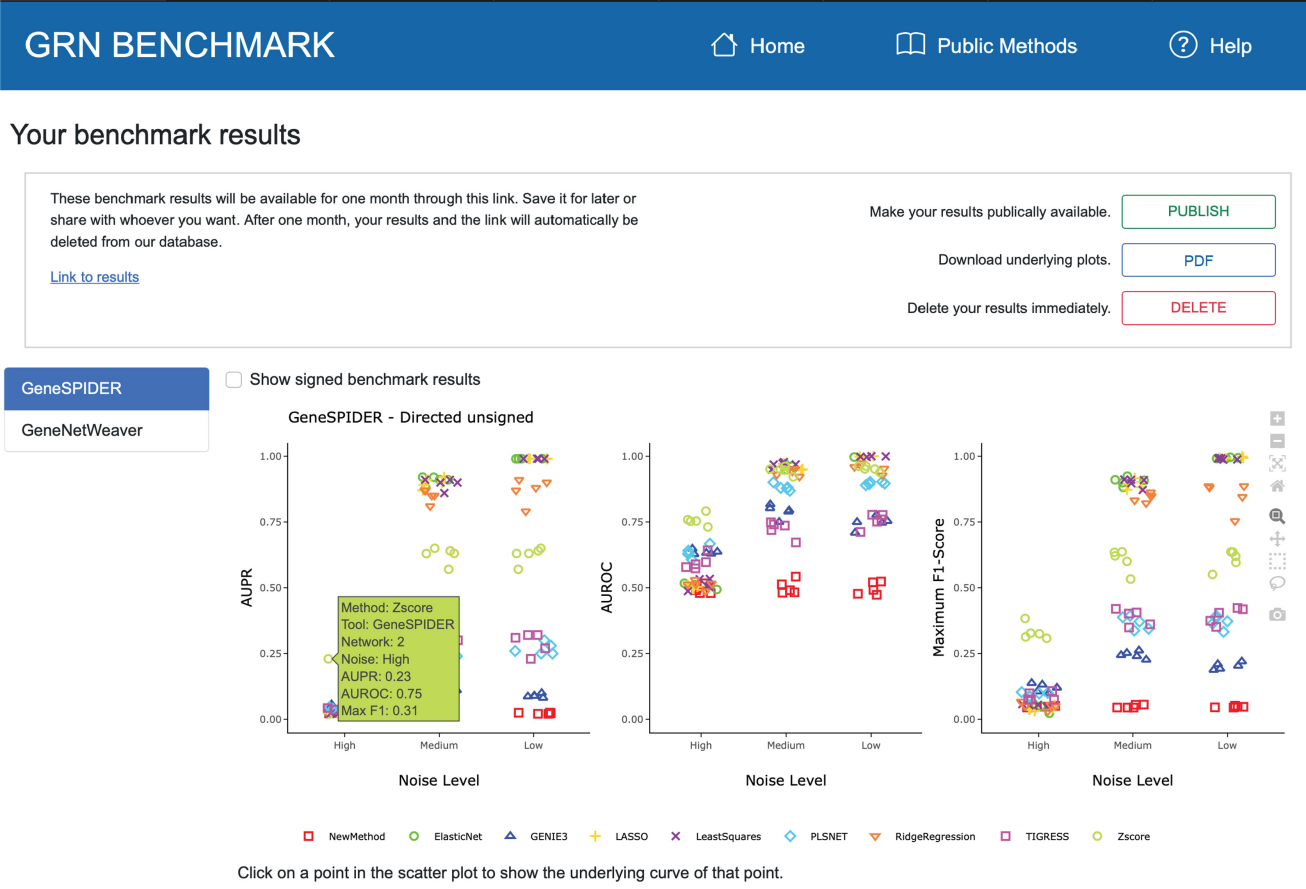
The interactive overview scatter plot summarizes the accuracies from all GRN inference methods in the database, i.e. all the public methods, along with the predictions submitted by a user if this is the case. Here ‘NewMethod’ was submitted, which contained random predictions. Three options are provided to the submitter: ‘PUBLISH’, ‘PDF’, and ‘DELETE’, to make the results public, to download a PDF of the results together with the public ones, and to delete the benchmark results immediately, respectively. On the left, one can select the current benchmark (GeneSPIDER or GeneNetWeaver), and next to this is a checkbox to switch between directed-unsigned and directed-signed results. Each plot shows the accuracy on the y-axis, as measured by AUPR, AUROC, or max F1-score. The benchmark has three different noise levels which are shown as categories on the x-axes in each plot. The left panel shows the popup hover window with benchmarking information for the marker under the mouse. Clicking on a marker displays its underlying curves (not shown). On the right is a column with buttons to control zooming, panning, and downloading.

GRNbenchmark takes approximately 5 minutes to complete the benchmarking for both tools, but this time doubles if the inferred GRNs contain the sign of the interaction since a separate signed benchmark is then executed. The accuracy of the inferred GRNs is visualized in a few different ways to provide a straightforward assessment of the given method's accuracy in comparison to the public methods. The overview scatter plot (Figure 2) is a three-panel interactive scatter plot for AUPR, AUROC and the maximum F1-score. Each marker in the first two panels corresponds to the area under the curve for a method, while the third panel shows the maximum F1-score across all sparsities. The underlying curves can be visualized by clicking on a marker in the scatter plot. Mouseover without a click on a marker in the scatter plot shows detailed information about a particular marker, including the method name, network name, and the exact values of the accuracies. The scatter plots are *Plotly* panels that provide multiple interactive functionalities. Each method can be turned on and off, allowing an easy investigation of results, and zooming/panning is possible to resolve crowded areas.

The left side of the results page contains the benchmark menu, where the available benchmarks are listed, currently GeneNetWeaver and GeneSPIDER. Clicking on a benchmark from this menu visualizes the results for the selected benchmark. By default, results for the directed unsigned benchmark are visualized, and an option for switching to the results from the directed signed benchmark is also available.

A noise-related trend, as expected, can be observed where the accuracy levels increase relative to decreasing noise in most cases, especially from high to medium noise levels. This increase is considerably less from medium to low, suggesting that almost all methods reach their maximum accuracy at the medium noise level. At medium and low noise levels, the accuracy of all inference methods are higher than the random performance, which is 0 for AUPR and 0.5 for AUROC. While some methods can reach almost perfect accuracy at these noise levels, some remain low. We observe that the winner of all methods is Z-score at high noise, while other methods such as least squares and LASSO may outperform it at medium and low noise levels. These interpretations of the accuracy results hold for benchmarks from both data generation tools, although the overall accuracy for the GeneNetWeaver benchmark is lower than for GeneSPIDER. The signed benchmark is only performed for methods which can infer a sign for an interaction, and the accuracy levels are about equal for both directed/unsigned and directed/signed, meaning that the true positives have their sign mostly inferred correctly. For more details of accuracy results, we refer the interested reader to ‘Public Results’ on the web server, which can either be inspected interactively or downloaded as PDF.

**Figure 3.**
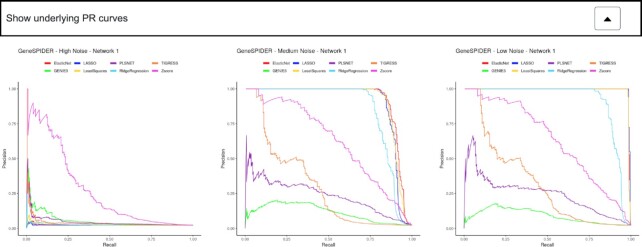
By expanding the ‘Show underlying PR curves’ tab, detailed precision-recall curves for each GRN is displayed. These curves were used for calculating the area under curve measures shown in the overview scatter plot. Likewise, curves for ROC and F1 versus sparsity curves are available for all GRNs.

In addition to viewing underlying curves for a single method and GRN by mouse click on a marker in the scatter, all curves for precision-recall (PR), receiver-operating-characteristic (ROC), and sparsity versus F1-score are shown for each dataset, combined for all methods (Figure 3). These curves are hidden under tabs called ‘Show underlying PR curves’, ‘Show underlying ROC curves’, and ‘Show underlying F1 curves’. Results visualized under tabs are no longer interactive, but can be saved and used separately for any purpose. Each of these plots can be separately downloaded in PNG format. The last tab is dedicated to the results table, where the exact values of all accuracies can be seen. A PDF report of all results is also generated and can be downloaded by the user.

### Storing user results

Once the benchmarking is complete, the results page is privately updated to the user by the addition of their method. A link is provided to the user, and kept active for 30 days. A benchmark report is generated in PDF format that can be downloaded as long as the link to the results page is active. All plots are downloadable separately in PNG format. For the overview scatter plots, this means that the visualization is no longer interactive.

In the results page, a user submitting inferred GRNs has two options available: ‘Delete’ and ‘Publish’. If the user proceeds with one of these options, the link to the results page is deactivated immediately, while no action keeps the link alive for 30 days. If the user clicks on the ‘Delete’ button, the results are immediately deleted, and if the user clicks on the ‘Publish’ button, it becomes one of GRNbenchmark's public methods.

## DISCUSSION

GRNbenchmark is the first online framework for benchmarking GRN inference methods. It provides a comprehensive selection of datasets, performs an automated benchmarking of the users’ predictions, and visualizes the accuracy results both interactively and in a format that is suitable for use in publications.

The data provided by GRNbenchmark are generated using different tools and at multiple levels of noise, which is often lacking in benchmarking data used in the field. Together with its automated and standardized benchmarking procedure this approach prevents biasing the data towards specific GRN inference methods. The interactive visualization of results that are stored in the link provided to the user enables an in depth assessment of methods’ accuracies and their underlying curves, with the three accuracy measures that are most commonly used in the field. The opportunity to publish the results of an inference method online is intended to help the method reach the entire GRN inference community and promote its use.

While GRNbenchmark provides a large collection of datasets with varying properties from two generation tools, it is currently limited to steady-state bulk data, excluding time-series and the recently emerging single-cell data. This, however, is not an inherent limitation since the framework allows addition of more benchmarks, *e.g*. for other experiment types, from other data generation tools, of different sizes, or with further variations of data properties. A benchmark for GRN inference from single-cell data would be interesting when those methods become more reliable, but at the moment they have very low accuracy (26). Considering the possible space for improvement, expanding the available data types is a future goal for GRNbenchmark.

GRNbenchmark performs the benchmarking in a directed manner, meaning that the source and target of the interaction matter in the resulting accuracy of the method. If the inference method also infers the sign of the interaction, the method is separately benchmarked against other methods that also infer the sign. However, GRNbenchmark is not suitable for methods inferring undirected networks, where the interaction between two genes is symmetrical. In such cases, for every true positive, a false positive is added, in addition to each false positive becoming two false positives, severely penalizing the method's accuracy. Therefore, inference methods that do not infer the direction of interactions, such as ARACNE (27) and CLR (Context Likelihood of Relatedness) (28), are not included in the publicly available methods, and we discourage users from submitting undirected (symmetrical) GRNs.

Considering the continuous development of new GRN inference methods, often with biases and limitations in the validation, GRNbenchmark provides a great advance to the field by its user-friendly online interface that provides a large amount of benchmarking data and an automated benchmarking procedure in comparison with other methods, with high quality interactive visualizations. GRNbenchmark is valuable for both developers of new GRN inference methods and researchers who want to select a method for a GRN inference problem based on either overall accuracy and robustness, or for a particular property such as noise level.

## DATA AVAILABILITY

The benchmarking data can be downloaded from https://grnbenchmark.org.

## References

[1] Emmert-Streib F. , Dehmer M., Haibe-Kains B. Gene regulatory networks and their applications: understanding biological and medical problems in terms of networks. Front Cell Dev Biol. 2014; 2:38. 25364745 10.3389/fcell.2014.00038 PMC4207011

[2] Price N.D. , Edelman L.B., Lee I., Yoo H., Hwang D., Carlson G., Galas D.J., Heath J.R., Hood L. Systems biology and systems medicine. Essentials of Genomic and Personalized Medicine. 2010; Elsevier Inc 131–141.

[3] Sonawane A.R. , Weiss S.T., Glass K., Sharma A. Network medicine in the age of biomedical big data. Front. Genet. 2019; 10:294. 31031797 10.3389/fgene.2019.00294 PMC6470635

[4] Huynh-Thu V.A. , Irrthum A., Wehenkel L., Geurts P. Inferring regulatory networks from expression data using tree-based methods. PLoS One. 2010; 5:e12776. 20927193 10.1371/journal.pone.0012776 PMC2946910

[5] Tibshirani R. Regression shrinkage and selection via the Lasso. J. R. Stat. Soc. Series B Stat. Methodol. 1996; 58:267–288.

[6] Haury A.-C. , Mordelet F., Vera-Licona P., Vert J.-P. TIGRESS: trustful inference of gene REgulation using stability selection. BMC Syst. Biol. 2012; 6:145. 23173819 10.1186/1752-0509-6-145 PMC3598250

[7] Guo S. , Jiang Q., Chen L., Guo D. Gene regulatory network inference using PLS-based methods. BMC Bioinformatics. 2016; 17:545. 28031031 10.1186/s12859-016-1398-6 PMC5192600

[8] Marbach D. , Costello J.C., Küffner R., Vega N.M., Prill R.J., Camacho D.M., Allison K.R., Kellis M., Collins J.J. DREAM5 Consortium et al. Wisdom of crowds for robust gene network inference. Nat. Methods. 2012; 9:796–804. 22796662 10.1038/nmeth.2016 PMC3512113

[9] Greenfield A. , Madar A., Ostrer H., Bonneau R. DREAM4: combining genetic and dynamic information to identify biological networks and dynamical models. PLoS One. 2010; 5:e13397. 21049040 10.1371/journal.pone.0013397 PMC2963605

[10] Madar A. , Greenfield A., Vanden-Eijnden E., Bonneau R. DREAM3: network inference using dynamic context likelihood of relatedness and the inferelator. PLoS One. 2010; 5:e9803. 20339551 10.1371/journal.pone.0009803 PMC2842436

[11] Tjärnberg A. , Morgan D.C., Studham M., Nordling T.E.M., Sonnhammer E.L.L. GeneSPIDER - gene regulatory network inference benchmarking with controlled network and data properties. Mol. Biosyst. 2017; 13:1304–1312. 28485748 10.1039/c7mb00058h

[12] Seçilmiş D. , Hillerton T., Morgan D., Tjärnberg A., Nelander S., Nordling T.E.M., Sonnhammer E.L.L. Uncovering cancer gene regulation by accurate regulatory network inference from uninformative data. NPJ Syst. Biol. Appl. 2020; 6:37. 33168813 10.1038/s41540-020-00154-6 PMC7652823

[13] Seçilmiş D. , Hillerton T., Nelander S., Sonnhammer E.L.L. Inferring the experimental design for accurate gene regulatory network inference. Bioinformatics. 2021; 37:3553–3559. 10.1093/bioinformatics/btab367 PMC8545292 33978748

[14] Hillerton T. , Seçilmiş D., Nelander S., Sonnhammer E.L.L. Fast and accurate gene regulatory network inference by normalized least squares regression. Bioinformatics. 2022; 38:2263–2268. 10.1093/bioinformatics/btac103 PMC9004640 35176145

[15] Pirgazi J. , Olyaee M.H., Khanteymoori A. KFGRNI: a robust method to inference gene regulatory network from time-course gene data based on ensemble kalman filter. J. Bioinform. Comput. Biol. 2021; 19:2150002. 33657986 10.1142/S0219720021500025

[16] Bellot P. , Olsen C., Salembier P., Oliveras-Vergés A., Meyer P.E. NetBenchmark: a bioconductor package for reproducible benchmarks of gene regulatory network inference. BMC Bioinformatics. 2015; 16:312. 26415849 10.1186/s12859-015-0728-4 PMC4587916

[17] Altenhoff A.M. , Garrayo-Ventas J., Cosentino S., Emms D., Glover N.M., Hernández-Plaza A., Nevers Y., Sundesha V., Szklarczyk D., Fernández J.M. et al. The quest for orthologs benchmark service and consensus calls in 2020. Nucleic Acids Res. 2020; 48:W538–W545. 32374845 10.1093/nar/gkaa308 PMC7319555

[18] Persson E. , Kaduk M., Forslund S.K., Sonnhammer E.L.L. Domainoid: domain-oriented orthology inference. BMC Bioinformatics. 2019; 20:523. 31660857 10.1186/s12859-019-3137-2 PMC6816169

[19] Hu Y. , Flockhart I., Vinayagam A., Bergwitz C., Berger B., Perrimon N., Mohr S.E. An integrative approach to ortholog prediction for disease-focused and other functional studies. BMC Bioinformatics. 2011; 12:357. 21880147 10.1186/1471-2105-12-357 PMC3179972

[20] Alliance of Genome Resources Consortium Harmonizing model organism data in the alliance of genome resources. Genetics. 2022; 220:iyac022. 35380658 10.1093/genetics/iyac022 PMC8982023

[21] Schaffter T. , Marbach D., Floreano D. GeneNetWeaver: in silico benchmark generation and performance profiling of network inference methods. Bioinformatics. 2011; 27:2263–2270. 21697125 10.1093/bioinformatics/btr373

[22] Zou H. , Hastie T. Regularization and variable selection via the elastic net. Journal of the Royal Statistical Society: Series B (Statistical Methodology). 2005; 67:301–320.

[23] Prill R.J. , Marbach D., Saez-Rodriguez J., Sorger P.K., Alexopoulos L.G., Xue X., Clarke N.D., Altan-Bonnet G., Stolovitzky G. Towards a rigorous assessment of systems biology models: the DREAM3 challenges. PLoS One. 2010; 5:e9202. 20186320 10.1371/journal.pone.0009202 PMC2826397

[24] Wickham H. ggplot2: elegant graphics for data analysis springer. 2016;

[25] Sievert C. Interactive Web-Based Data Visualization with R, plotly, and shiny. 2020; 1st edn Chapman and Hall/CRC 10.1201/9780429447273.

[26] Pratapa A. , Jalihal A.P., Law J.N., Bharadwaj A., Murali T.M. Benchmarking algorithms for gene regulatory network inference from single-cell transcriptomic data. Nat. Methods. 2020; 17:147–154. 31907445 10.1038/s41592-019-0690-6 PMC7098173

[27] Margolin A.A. , Nemenman I., Basso K., Wiggins C., Stolovitzky G., Favera R.D., Califano A. ARACNE: an algorithm for the reconstruction of gene regulatory networks in a mammalian cellular context. BMC Bioinformatics. 2006; 7(Suppl.1):S7. 10.1186/1471-2105-7-S1-S7 PMC1810318 16723010

[28] Faith J.J. , Hayete B., Thaden J.T., Mogno I., Wierzbowski J., Cottarel G., Kasif S., Collins J.J., Gardner T.S. Large-scale mapping and validation of escherichia coli transcriptional regulation from a compendium of expression profiles. PLoS Biol. 2007; 5:e8. 17214507 10.1371/journal.pbio.0050008 PMC1764438

